# The Role of Oestrogen and Other Hormones in the Pathophysiology and Treatment of Schizophrenia

**DOI:** 10.1155/2012/540273

**Published:** 2012-02-19

**Authors:** Emily Hayes, Emorfia Gavrilidis, Jayashri Kulkarni

**Affiliations:** Monash Alfred Psychiatry Research Centre, The Alfred Hospital and Monash University School of Psychology, Psychiatry and Psychological Medicine, Melbourne, VIC 3004, Australia

## Abstract

The theory that many serious mental illnesses, in particular psychoses such as schizophrenia, may have a significant hormonal aetiological component is fast gaining popularity and the support of scientific evidence. Oestrogen in particular has been substantially investigated as a potential mediator of brain function in schizophrenia. Epidemiological and life-cycle data point to significant differences in the incidence and course of schizophrenia between men and women suggests a protective role of oestrogen. *In vitro* and *in vivo* preclinical research confirms oestradiol's interactions with central neurotransmitter systems implicated in the pathogenesis of schizophrenia, while results from randomised controlled trials investigating the antipsychotic potential of oestrogen have been positive. Research into other neuroactive hormones with possible effects on mental state is a rapidly evolving field that may hold new promise. Given that schizophrenia and related psychoses are pervasive and debilitating conditions for which currently available treatments are often only partially effective and entail a high risk of serious side-effects, novel therapeutic strategies are needed. The literature reviewed in this paper suggests that hormones such as oestrogen could be a viable option, and it is hoped that with further research and larger trials, the oestrogen hypothesis can be translated into effective clinical practice.

## 1. Introduction

Over a century ago, the father of modern psychiatry Emil Kraepelin first implicated an imbalance of sexual hormones in the aetiology of “dementia praecox” [1], and reports of gonadal dysfunction in psychotic patients have also been well documented since this time [2]. However it is only with recent scientific advances that the considerable effects of reproductive hormones on central nervous system functioning and mental health have come to light. Such evidence has led multiple researchers [3–6] to investigate the role of oestrogen in the pathogenesis of psychosis and propose the “oestrogen protection hypothesis” and the “hypothesis of hypoestrogenism” as possible explanations for gender differences in schizophrenia.

This paper provides a summary of the current literature with the intention of highlighting the likely role and clinical importance of oestrogen and other reproductive hormones in psychotic illness, in particular in the field of women's mental health.

## 2. Case Study

Miss R was a 52-year-old woman with a history of schizophrenia which began after the birth of a child she gave up for adoption in her late twenties. She had been treated with olanzapine 15 mg oral per day for the past 10 years and had been very well, with no hospitalisations. Miss R worked as a sales assistant and lived in her own apartment. However from the age of 50, her mental state deteriorated significantly and she experienced auditory hallucinations and paranoid delusions. She believed that the CIA had implanted a microchip into her brain and that she was under constant surveillance. She described hearing several spies talking about her. Miss R's quality of life suffered greatly. She lost her job, was unable to live independently, and financially existed on welfare payments. Miss R lived in a derelict boarding house and had repeated hospitalisations with no real improvement. Miss R did not respond to a succession of treatment trials with a variety of second generation antipsychotic medications. She was treated with adjunctive estradiol (100 mcg transdermal estradiol) and had a progesterone secreting intrauterine device (IUD) inserted. The hormone treatment was added to her antipsychotic treatment—which had been risperidone 6 mg oral per day for the past two months. Within one week of adding hormone treatment, she made a dramatic improvement in her mental state and had no auditory hallucinations. The paranoid delusions also resolved within two weeks of adding hormone treatment to her antipsychotic medication. Over a period of months, Miss R was able to find herself better accommodation and some part-time employment. She has remained well—both mentally and physically after 4 years, still taking risperidone plus 50 mcg transdermal estradiol plus the IUD.

## 3. Epidemiological Findings

While the nature of schizophrenia was previously perceived to be similar between men and women [7], it is now widely accepted that schizophrenia is a sexually dimorphic disease.

To begin with, the incidence of schizophrenia in men is consistently observed to be approximately 1.5 times higher than the incidence rate for women [8]. There are also significant differences in age distribution of the illness between males and females: men reliably present on average four years earlier than their female counterparts [6, 9], while women display a second spike in incidence between the ages of 45 and 54 [2, 6]. Thus there is a male predominance in incidence in the early twenties, but a female predominance in older middle age [10]. Two recent large-scale studies [11, 12] of individuals with psychotic illness have also found that premenopausal women tend to experience a more benign course of illness than men, displaying less severe levels of psychopathology and disability, and higher levels of insight, functioning, and response to antipsychotic medication. This holds true until the age of 45, when the resurgence in incidence in women is associated with unusually severe symptomatology and a need for higher doses of antipsychotic medications [13]. It is widely believed that this unique course of illness in older women is related to falling levels of oestrogen during the menopause, the hormone having been protective against psychosis up until this time, that is, the *oestrogen protection hypothesis*.

## 4. Life-Cycle Findings

The oestrogen protection theory is further supported by evidence from life-cycle studies in females. For example, women have a 20-fold increased risk of suffering a first episode or relapse of psychosis during the postpartum period when oestrogen levels plummet dramatically [14], whereas chronic psychoses tend to improve during pregnancy when oestrogen is extremely high [13].

Similarly, in women with schizophrenia, psychotic symptoms appear to fluctuate throughout the menstrual cycle, a trend first noted by Krafft-Ebing in the late 1800s: the so-called “menstrual psychosis” [15]. Modern-day research shows that during the menstrual cycles of female schizophrenia patients, the high-oestrogen luteal phase is associated with significant improvements in psychopathology and functioning compared to the low-oestrogen follicular phase [16–19].

In short, these epidemiological and life cycle observations offer compelling evidence to suggest that oestrogen protects women against severe psychosis during their childbearing years, and that oestrogen withdrawal of any kind may be able to induce psychosis in vulnerable women [20].

## 5. Preclinical Findings

Oestrogen is well known to have significant actions in the central nervous system (CNS) beyond its primary endocrine and reproductive functions, so much so that it has been referred to as “nature's psychoprotectant” [21]. Indeed, oestrogen receptors can be found in abundance in multiple extra-hypothalamic regions throughout the brain, in particular the limbic system, basal ganglia, cerebellum, and many areas of the cerebral cortex [22, 23]. Through classical genomic and rapid nongenomic interactions with these receptors, oestrogen functions as a “neuroactive steroid”, influencing signalling pathways and neurodegenerative processes within the CNS [24]. Recent biochemical and animal research has helped enhance our understanding of the neuromodulatory and neuroprotective properties of oestrogenic compounds.

### 5.1. Neurotransmitter Modulation

While the historical dopaminergic hyperactivity hypothesis of schizophrenia endures, it is now widely accepted that other major neurotransmitter systems such as serotonin and glutamate are also involved in the pathophysiology of the disorder [25, 26]. The majority of antipsychotic drugs share the property of dopamine D_2_ receptor antagonism, while second generation antipsychotics also interact with serotonin 5-HT_2A_ and 5-HT_1A_ receptors [27]. Oestradiol has been found to significantly influence the dopaminergic, serotonergic, and glutamatergic systems, meaning that it may have properties similar to those of atypical antipsychotics [22, 28].

The effects of oestrogen on the dopaminergic system are thought to be highly complex and not well understood, with different studies reporting extensive variations in the direction, extent, and specificity of oestrogen-dopamine interactions [26, 29]. A number of studies involving ovariectomised (OVX) rats, however, have found an oestrogen treatment-associated increase in D_2_ receptor density in the striatum [26]. It has been hypothesised that this could be a compensatory response to an oestrogen-induced reduction in DA levels [22], possibly through enhanced action of the DA transporter (DAT). A recent study by Chavez et al. [29] found that OVX rats had considerably reduced DAT in the nucleus accumbens compared to intact rats, the density of which then increased significantly following chronic oestradiol treatment.

Oestrogen has similarly been found to have considerable effects on the serotonergic system at multiple levels. Lokuge and colleagues [30] recently reviewed extensive rodent and primate data on the CNS effects of oestrogen to conclude that oestradiol decreases the activity of monoamine oxidase, increases the activity of tryptophan hydroxylase, manipulates expression of the serotonin transporter, downregulates 5-HT_1A_ receptors, and upregulates 5-HT_2A_ receptors.

Hypoglutamatergic neurotransmission has also been implicated in the pathophysiology of schizophrenia given the observation that glutamate NMDA receptor antagonists such as phencyclidine induce a psychomimetic state in animals and humans [31]. Oestradiol is known to upregulate NMDA receptors, change their subunit configuration, and increase NMDA agonist binding in the rat brain [32], which could presumably help reverse hypoactive glutamatergic functioning in schizophrenia.

A number of recently published studies using animal models of schizophrenia have confirmed oestrogen's involvement with central neurotransmitter mechanisms and resultant antipsychotic-like activity. Gogos and colleagues mimicked a psychotic state in OVX rats by administering a D_2_ receptor agonist, a 5-HT_1A_ agonist, and a NMDA receptor antagonist, respectively [33, 34]. Chronic oestradiol treatment was able to reverse the psychotic endophenotype in all three instances, strongly suggesting that oestrogen's antipsychotic properties stem from its interactions with dopamine, serotonin, and glutamate. Work by Arad and Weiner [35, 36] supplements these findings and further exhibits oestradiol's antipsychotic potential. They found that oestradiol can ameliorate an amphetamine-induced psychotic state in both OVX and intact female rats as effectively as clozapine or haloperidol; however OVX rats required significantly higher doses of each compound to achieve this [35]. This lends credence to the oestrogen protection hypothesis and its consequences for the menopause as discussed earlier. Also, ineffective low doses of clozapine and haloperidol regained antipsychotic efficacy when combined with a low dose of oestradiol [35], which could have important implications for augmentation of antipsychotic drugs in schizophrenic women. A follow-up study by the same authors importantly demonstrates that oestradiol has efficacy reversing psychotic behaviours in both female and male rats [36], raising the possibility of clinical use of oestrogen in both sexes.

### 5.2. Neuroprotection

Schizophrenia is considered by many to be at least in part a progressive neurodegenerative disorder [37, 38]. Numerous anatomical abnormalities have been reported in the brains of schizophrenia patients including reduced grey and white matter volume in multiple brain regions, ventricular enlargement, and abnormalities of the medial temporal lobe, prefrontal cortex, and cerebellum [38–40]. Abnormal cytoarchitecture is also common with neuronal soma and neuropil volume reductions, irregular synaptic organisation, ectopic neurons, and decreased expression of neurotrophic factors [37, 41].

Oestrogen is known to have diverse neuroprotective properties that could be of particular relevance to its ability to mediate the onset and course of neuropathology in schizophrenia. Recent *in vitro* and *in vivo* research has confirmed that oestrogenic compounds can protect brain cells against injury from excitotoxicity, oxidative stress, inflammation, ischaemia, and apoptosis [42–46]. They can also enhance neurogenesis, angiogenesis, synaptic density, plasticity and connectivity, axonal sprouting and remyelination, and expression of neurotrophic factors [2, 47–50]. Recent research suggests that the psychoprotective properties of oestrogens might arise from their preservation and enhancement of neuronal mitochondrial function, as mitochondria are responsible for regulating the viability and death of neurons [51] and may be defective in the brains of individuals with schizophrenia [52].

## 6. Clinical Findings

### 6.1. The Hypothesis of Hypoestrogenism

In her recent review of the literature, Markham speculates that, given the apparent psychoprotective actions of oestrogen in women, “those women who do end up developing schizophrenia are at greater risk in part because for some reason their oestradiol levels are abnormally low” [9]. This hypothesis was first introduced at the beginning of the 20th century when early researchers such as Kretschmer [53] observed signs of “insufficient functioning of the sexual glands” and “chronic hypoestrogenism” in female schizophrenia patients.

More recent work has confirmed this early theory of gonadal dysfunction in women with schizophrenia, with menstrual irregularities, anovulation, infertility, signs of hyperandrogenism, and reduced circulating levels of oestradiol, progesterone, follicle stimulating hormone, and luteinizing hormone frequently observed (see Riecher-Rossler and Kulkarni, 2011 [2], and Riecher-Rossler, 2002 [13], for reviews). Most interestingly, this historical finding has recently also been extended to men, with separate research groups observing not only significantly reduced circulating oestradiol concentrations in acutely psychotic men compared to controls, but also an inverse correlation between oestrogen levels and negative symptoms [54, 55]. 

However, it is still not entirely clear whether this gonadal dysfunction precedes or follows the onset of psychosis—that is, whether it is a causative or a resulting factor.

The potential for both the chronic emotional stress associated with a psychiatric disorder such as schizophrenia and antipsychotic medication-induced hyperprolactinaemia to disrupt the hypothalamic-pituitary-gonadal (HPG) axis [56, 57] is a possible way in which hypoestrogenism could be a consequence of schizophrenia. However, several lines of evidence contest this. To begin with, schizophrenia-associated gonadal hypofunction in women was observed by the likes of Kraeplin and Kretschmer long before the introduction of neuroleptic drugs, and other psychiatric diagnoses that also entail a high level of stress appear not to be associated with as great a degree of HPG axis perturbation as schizophrenia [58, 59]. Furthermore, Riecher-Rossler et al. found that a history and signs of “severely disturbed” gonadal function were significantly more common in women admitted for *first episode* psychosis than controls [60], while Maric and colleagues report that bone mass density—an indicator of chronic oestrogen deficiency—is significantly lower in women with first onset schizophrenia than matched controls [61]. Finally, multiple researchers have noted hypoestrogenism in women with schizophrenia irrespective of prolactin level [58, 62, 63]—that is, not just in those women with hyperprolactinaemia.

These findings would seem to indicate the presence of hypoestrogenism prior to the onset of psychosis and independent of neuroleptic-induced endocrine disturbances or emotional stress. More research is needed to further explore the possibility that underlying oestrogen deficiency could be involved in the initial pathogenesis of psychosis.

### 6.2. Oestradiol as Treatment: Intervention Studies

Given the evidence in support of both the oestrogen protection hypothesis and the hypothesis of hypoestrogenism, it is not surprising that oestradiol has been targeted as a potential therapeutic agent for schizophrenia. And although two recent reviews in 2005 and 2007 concluded that adjunctive oestrogen does not seem to be any more advantageous than placebo in the treatment of schizophrenia [64, 65], more recent work has yielded some promising results. 

In a randomised sample of 102 women diagnosed with a psychotic illness, our research group found that adjunctive treatment with 100 *μ*g per day of transdermal oestradiol for 28 days led to significant improvements in total Positive and Negative Syndrome Scale (PANSS) scores (*P* = 0.002), positive symptoms (*P* = 0.005), general psychopathology (*P* = 0.01), and cognition (*P* = 0.01) when compared with antipsychotic medication alone [66]. These findings are consistent with the results of an earlier, smaller randomised controlled trial by Ahkondzadeh and associates who also reported significant reductions in total, positive symptom and general psychopathology PANSS scores for schizophrenic women treated with haloperidol plus ethinyl oestradiol compared to women treated with haloperidol plus placebo [67].

Most recently, our research group has piloted the use of adjunctive oestrogen therapy in men with schizophrenia [68], as well as adjuvant use of the selective oestrogen receptor modulator (SERM), raloxifene, for postmenopausal psychosis [69]. In a two-week randomised controlled trial we treated 53 men with either 2 mg oral oestradiol valerate or placebo. The oestradiol group displayed a significantly faster improvement in general psychopathology than the placebo group (*P* < 0.05) with no increase in adverse side-effects, providing the first clinical indication that oestrogen could be a viable treatment adjunct in men as well as women. In a preliminary dose-finding study, we also trialled 120 mg augmentation per day of oral raloxifene for 12 weeks in postmenopausal women with schizophrenia. SERM therapy resulted in significantly greater recovery in total (*P* < 0.0005) and general psychopathology (*P* < 0.0005) PANSS scores than was observed with placebo. Importantly, these results have been replicated in a similar study conducted by Usall et al. [70], who observed significant improvements in all PANSS subscales in postmenopausal women treated with adjunctive raloxifene compared to placebo (*P* < 0.05). These findings are also consistent with a small, earlier study by Good et al. [71] who found that hormone replacement therapy in postmenopausal women with a psychotic disorder led to a significant improvement in negative symptoms over six months. Furthermore, other researchers have also reported significant improvements in elements of neuropsychological and cognitive performance for women with schizophrenia treated with oestradiol as compared to placebo [72, 73].

While some other studies [74, 75] have failed to detect a benefit of adjunctive oestrogen over placebo in the management of schizophrenia, these results should be interpreted with caution. For example, Louzã et al. [74] administered conjugated oestrogens to their participants, as opposed to 17-b-oestradiol, while Bergemann et al. [75] administered oestradiol in conjunction with a synthetic progestin. Conjugated oestrogens do not share the same potency as 17-b-oestradiol in the brain, while administration of a progestin in combination with oestradiol is often believed to attenuate any positive effects of oestradiol on mental state [2, 9], which could explain the negative findings in these studies.

## 7. Other Hormones

Subsequent to the ever-expanding knowledge base on the CNS effects of oestrogen, interest in other neuroactive steroids with neuromodulatory properties and therapeutic potential has intensified [76].

The relationship between androgens and mental state seems particularly complicated. Animal evidence suggests that testosterone may be propsychotic, given that administration of testosterone significantly enhanced an NMDA antagonist-induced psychotic state in OVX rats [34]; however most of the research to date into androgens and mental state has focused on the testosterone precursors dehydroepiandrosterone (DHEA) and DHEA-sulfate (DHEA-S). DHEA(S) is neuroprotective in the rodent brain [77], and differences in DHEA(S) blood levels between psychotic patients and healthy controls are widely reported; however the direction of these differences is far from consistent [78]. Results from clinical studies trialling DHEA(S) as an augmentation strategy have been similarly contradictory, with some studies finding a modest treatment effect and others reporting no superiority over placebo [78]. Further research is needed.

Pregnenolone and its metabolites pregnenolone sulfate and allopregnanolone seem more promising. In addition to also possessing neuromodulatory and neuroprotective properties, these neurosteroids exert positive effects in rodent models of cognition and psychosis [79]. Serum levels of pregnenolone have been found to be lower in patients with schizophrenia than in healthy controls, and antipsychotic medications significantly increase pregnenolone levels in the brain [78]. A review of three small pilot studies investigating pregnenolone as an adjunctive intervention for patients with schizophrenia reports that pregnenolone was able to improve psychotic and cognitive symptoms [79], demonstrating the exciting potential of this compound. Similarly, progesterone itself has come under scrutiny in the topic of psychotic illness given that, like oestradiol, levels fluctuate throughout the menstrual cycle and drop dramatically after partum. Some researchers have found lower plasma progesterone levels in schizophrenia patients compared to healthy controls [80], while many others report antipsychotic-like properties of progesterone in behavioural paradigms of psychosis [81], in both animals [82] and humans [83].

Most recently, oxytocin has also emerged as possibly influencing mental state after one study found that higher peripheral oxytocin levels were associated with decreased symptom severity in women with chronic schizophrenia [17], and another study demonstrated efficacy of intranasal oxytocin as an adjunctive therapy in a randomised, cross-over sample of fifteen schizophrenia patients [84].

## 8. Discussion

The oestrogen protection hypothesis and hypothesis of hypoestrogenism may have important implications for clinical practice, especially in the area of women's mental health.

To begin with, the clinician must be aware of the possibility of an exacerbation or new onset of psychotic illness during the perimenopause, especially in women with a history of deteriorations in mental state during the menstrual cycle or puerperium, as such women seem to be particularly vulnerable to fluctuations in the hormonal milieu. It is these women who might benefit most from oestrogen augmentation therapy, especially considering the additional benefits of oestrogen replacement during the menopause. This clinical scenario is illustrated poignantly in the case of Miss R, detailed earlier.

While oestrogen has proven neuroprotective and antipsychotic effects, its long-term safety for use as an adjunctive treatment in schizophrenia is unclear given its stimulating effects on peripheral tissues such as the breast and endometrium [85]. Hence recent research into SERMs is particularly important. SERMs have been found to share the neuroprotective and neuromodulatory actions of oestradiol in the CNS [42, 86] but have tissue-specific effects on peripheral oestrogen receptors [87]. Raloxifene, for example, has agonist actions in the brain but antagonist actions in the breast and endometrium [85], and the first clinical evidence of raloxifene's potential as a safe and effective augmentation therapy in women with schizophrenia is beginning to emerge [69, 70].

Finding safe and effective treatments to augment antipsychotic medication and thus minimise their dosage is particularly important in women being treated for schizophrenia. Women are more susceptible to antipsychotic-induced hyperprolactinaemia, which can result in serious sequelae such as early menopause, osteoporosis, and perhaps even breast cancer [9, 88]. Raloxifene could thus be an ideal option for augmentation considering that it actually preserves bone density and has anticancer properties in breast tissue.

Finally, that oestrogen has been shown in multiple studies to significantly improve general psychopathologic symptoms such as depression, anxiety, insight, and cognition is of particular importance to the treatment of schizophrenia and not to be underestimated. Improvement in these domains independent of changes in psychotic symptomatology can dramatically improve a patient's quality of life, engagement in services, compliance with treatment, response to stressors, and overall psychosocial functioning [89].

## 9. Conclusion

The case of Miss R highlights the real clinical relevance and therapeutic potential of oestrogen in schizophrenia, lending credence to the theories discussed in this paper. Study of the complex relationship between hormones—in particular oestrogen—and neuropsychological functioning is a rapidly progressing field, and recent research has significantly enhanced our knowledge about the nature of the connection between oestrogen and schizophrenia. Oestrogen may be effective not only in treating the symptoms of schizophrenia but also in mediating the underlying neurochemical and neuropathological abnormalities; however more work needs to be done to determine the ideal delivery, dose, and duration of treatment to achieve this. Thus research into both the biological underpinnings of neuroactive steroids' mechanisms of action in the brain and the therapeutic potential of different hormonal compounds must persist, so clinical practice in the diagnosis and management of psychotic conditions can continue to evolve.
